# The impact of anxiety, depression, and social support on the relationship between HIV-related stigma and mental health-related quality of life among Chinese patients: a cross-sectional, moderate-mediation study

**DOI:** 10.1186/s12888-023-05103-1

**Published:** 2023-11-08

**Authors:** Yushu Zhang, Chengliang Chai, Jianjing Xiong, Lin Zhang, Jinlei Zheng, Zhen Ning, Ying Wang

**Affiliations:** 1 School of Public Health, Fudan University, Shanghai, China; 2 NHC Key Laboratory of Health Technology Assessment, Fudan University, Shanghai, China; 3 Department of AIDS/STDs Control and Prevention, Zhejiang Provincial Center for Diseases Control and Prevention, Zhejiang Province, Hangzhou, China; 4 Jing’an District Center for Disease Prevention and Control, Shanghai, China; 5 Center for Disease Control and Prevention, 10, East Section of Taihao Road, Henan Province, Zhoukou, 466000 China; 6 Zhejiang Provincial Center for Disease Control and Prevention, Zhejiang Province, Hangzhou, China; 7 Division of Tuberculosis and AIDS Control and Prevent Shanghai Municipal Center for Disease Control and Prevention 1380, Zhongshan Xi Road, Shanghai, 200336 China; 8 Department of Social Medicine, School of Public Health, Fudan University, Shanghai, 200032 China

**Keywords:** HIV-related stigma, Depression, Anxiety, Mental health-related quality of life, Social support

## Abstract

**Background/objective:**

People living with HIV (PLWH) are prone to mental health problems and evidence indicates that HIV-related stigma can negatively impact mental health-related quality of life. This study explored potential mechanisms between HIV-related stigma and mental health-related quality of life, specifically whether anxiety or depression mediates, and whether social support moderates, the relationship.

**Method:**

A total of 1197 Chinese PLWH participated in the study. The Berger HIV Stigma Scale, the Hospital Anxiety and Depression Scale (HADS), the 12-item Brief Health Survey (SF-12), and the Multidimensional Scale of Perceived Social Support (MSPSS) were employed.

**Results:**

HIV-related stigma was negatively associated with mental health-related quality of life. Anxiety and depression partially mediated the relationship between HIV-related stigma and mental health-related quality of life; social support played a moderating role.

**Conclusions:**

The mental health-related quality of life in PLWH was shown to be indirectly affected by HIV-related stigma through anxiety and depression in China. The negative impact of HIV-related stigma decreased with increased social support.

## Introduction

AIDS remains a global public health problem, with 38.4 million people living with HIV (PLWH) worldwide as of 2021 [1]. Although antiretroviral therapy (ART) can improve the life expectancy of PLWH [2], they face comorbidities that can severely affect health-related quality of life (HRQoL). A study conducted in the UK compared the HRQoL of PLWH with that of the general population and found a substantial disparity, with PLWH experiencing significantly lower HRQoL [3]. Growing evidence suggests that various factors, including HIV-related stigma [4], depression [5], anxiety, and spiritual beliefs concerning their disease and medications, influence the mental and physical dimensions of HRQoL in PLWH [6, 7]. HRQoL encompasses multiple aspects of physical health, mental well-being, and social functioning [5]. PLWH also exhibit a high prevalence of mental health problems [8], and changes in HRQoL may persist throughout their lives [9]. Despite this, the specific factors influencing mental health-related quality of life (MHRQoL), a crucial component of HRQoL, and the mechanisms by which they impact the psychological aspects of HRQoL have received limited attention in existing literature. Therefore, it is imperative to identify the factors that influence MHRQoL and explore the underlying mechanisms in order to facilitate improvements in the mental health and overall HRQoL of PLWH.

Since the beginning of the AIDS epidemic, HIV-related stigma has posed significant challenges for PLWH, not only leading to mental health and substance abuse issues [10], but also amplifying social inequalities, affecting access to and uptake of key resources [11]. Stigma has also been found to adversely affect the psychological dimension of HRQoL among PLWH [12, 13]. While previous studies have primarily focused on the impact of stigma levels on MHRQoL, there is limited research examining whether the influence of HIV-related stigma on mental health is mediated or moderated by other factors.

Anxiety and depression are critical factors affecting MHRQoL. It is common for PLWH to have anxiety and depression, with prevalence rates higher than 40% and 60%, respectively, in China [14]. Studies have found that people who experience any level of HIV-related stigma are significantly more likely to experience mild, moderate, and severe anxiety [15]. HIV-related stigma also exacerbates the risk of depression, a factor that has been shown to mediate the relationship between stigma and pain among HIV-infected women in the United States [16]. Overall, HIV-related stigma can make it more likely that PLWH will experience negative emotions such as anxiety and depression, emotions that, in turn, can have a notable impact on their MHRQoL.

Social support may help PLWH cope with HIV. Developing multiple social support resources may protect HIV-positive adolescents from experiencing poor mental health and may have a direct protective effect on depressive symptoms [17]. Moreover, social support buffers the relationship between stigma and HIV symptoms [18], with some inverse effect on stigmatization. However, one study found that social support did not moderate the association between enacted stigma and depression in PLWH [19]. Improving mental health-related quality of life (MHRQoL) in individuals experiencing HIV-related stigma may not be solely achieved by increasing social support [20]. Despite the limited moderating effect, social support may play a role in moderating the relationship between stigma and quality outcomes among PLWH.

HIV-related stigma has been recognized as having a direct impact on MHRQoL of PLWH; anxiety, depression, and social support are also significantly associated. Yet, how these psychosocial factors interact with MHRQoL has rarely been explored fully. Therefore, in order to better understand the possible mechanisms between these factors, this study hypothesized that: (1) HIV-related stigma is negatively associated with MHRQoL; (2) HIV-related stigma is positively associated with anxiety and depression; (3) anxiety and depression are negatively associated with MHRQoL; (4) anxiety and depression mediate the relationship between HIV-related stigma and MHRQoL; (5) social support moderates the relationship between anxiety and depression-mediated HIV-related stigma and MHRQoL.

## Methods

### Participants and procedure

The cross-sectional study was conducted from November 2017 to November 2018 in three regions of China: Shanghai, Henan, and Zhejiang. A multi-stage, stratified, random-sampling method based on the duration of antiretroviral treatment, age, and gender was used to select 1331 PLWH. Study eligibility criteria included (a) age ≥ 18 years; (b) diagnosis of HIV and infection for at least one year; (c) at least three months as a highly active antiretroviral therapy (HAART) recipient; (d) voluntary participation in the study after informed consent; (e) clear consciousness and language and ability to cooperate with the instructions and requirements of the study.

The study was conducted by local Centers for Disease Control and Prevention (CDC) agencies in the three regions. Relevant questionnaires were completed face-to-face after eligible participants signed informed-consent forms. Following the sampling plan, a total of 1331 eligible PLWH were contacted. After removing participants who were unable to complete the questionnaire for various reasons, a valid sample of 1273 PLWH was generated. In further analysis, the number of participants with some variables missing was 76 and they were removed, resulting in a final sample size of 1197 and a validity rate of 89.93%.

## Measures

### Demographic variables

Data related to the following sociodemographic variables were collected: age (≤ 44, 45–60, ≥ 60 years), sex (male/female), ethnicity (Han/other), religion (faith/none), marital status (single/married/divorced/widowed), employment status (employed/unemployed), and duration of treatment (< 1, 1–4, 5–9, ≥ 10 years).

### HIV-related stigma

Participants’ HIV-related stigma was assessed using the Berger HIV Stigma Scale [21], which consists of four subscales: personalized stigma (18 items), disclosure concerns (12 items), negative self-image (9 items), and concern with public attitudes (12 items), for a total of 40 items. All four dimensions were included in the measurement in this study. Participants utilized a Likert scale to respond to each item, scored 1–4 with a total score range of 40–160; a higher score indicated a greater sense of HIV-related discrimination. In this study, the Cronbach α coefficient of the questionnaire was 0.860 and the internal consistency coefficients of the four subscales were 0.929, 0.762, 0.801 and 0.922, respectively, indicating that the reliability was good.

### Anxiety and depression

Anxiety and depression were assessed by the Hospital Anxiety and Depression Scale (HADS) [22]. The HADS is a 14-item self-report screening scale originally developed to indicate the possible presence of anxiety and depressive states in non-psychiatric outpatient settings. Many studies have shown the scale to have good psychometric properties [23, 24]. The HADS consists of two subscales measuring anxiety (7 items) and depression (7 items), with items rated on a 4-point scale, yielding a potential maximum sub score of 21 each for depression and anxiety. The scale contains 14 questions such as I feel tense (or painful) and I can sit comfortably and easily. In this study, the Cronbach α for the scale was 0.682.

### Social support

The Multidimensional Scale of Perceived Social Support (MSPSS) [25] was employed to assess social-support level. The scale contains 12 items that measured the social support participants received in three dimensions: family, friends, and significant others. Specific aspects of the scale, such as my family's ability to give me practical and concrete help, my ability to discuss my problems with friends, etc. A seven-point (1 = strongly disagree to 7 = strongly agree) Likert scale scoring method yielded total scores ranging from 12 to 84 across the 12 questions. Higher scores indicated greater total social support from the three dimensions. Zimet et al. [25] tested the MSPSS, finding an internal consistency of 0.880 with a high confidence level.

### Mental health-related quality of life

Participant MHRQoL was assessed by the 12-item Brief Health Survey (SF-12) [26], a scale that has been accurately validated for use in Europe [27]. Consisting of 12 items, the survey assessed participant health in 8 areas: physical functioning (PF), role physical (RP), bodily pain (BP), general health (GH), vitality (VT), social functioning (SF), role emotional (RE), and mental health (MH). The eight areas were divided into two scales: the Physical Component Summary (PCS) and Mental Component Summary (MCS). This study focused on the MCS, which has a Cronbach's alpha of 0.720 [28], with scores ranging from 0 to 100. Higher scores indicated better MHRQoL.

### Statistical analyses

All data analyses were performed using SPSS v. 26 [29]. Results were considered significant if p < 0.05. If data were missing for a variable, the listwise delete method was used and the participant’s data was excluded from the analysis. The analyses controlled for covariates (age, gender, religion, employment status, marital status, and time of treatment), as they have all been shown to have an effect on mental health in PLWH [30–33]. However, Marital status and Time of treatment were excluded from the final model because they were not significantly associated with MHRQoL in our sample.

First, a partial correlation analysis was performed to examine the correlation between the variables of interest. The mediation model and the moderated mediation model were analyzed using process macros in SPSS [34]. All continuous variables were centralized. The hypothesized mediation model was tested using model 4 in the process macro, which consists of an independent variable, a dependent variable, and a mediating variable. That is, HIV-related stigma as the independent variable, anxiety and depression as mediating variables, and mental health as the dependent variable. Next, the established moderated mediation model was tested using model 59, which is an additional moderating variable compared to Model 4. In this study, social support was added as a moderator variable. Furthermore, simple slope calculations were performed on moderated models to test the significance of the moderated slopes. Finally, the study calculated a 95% confidence interval (CI) based on bootstrap estimation [35] with 5000 bootstrap samples.

## Results

### Sample characteristics

Table 1 presents the demographic characteristics of the study’s participants, 1273 PLWH. The mean age of the participants was 45.39 years (SD = 13.64). Most were male (74.9%), of Han ethnicity (95.7%), and had no reported religion (83.9%). Of the PLWH participants, 45.1% were married and 52.8% were employed.

**Table 1:** Characteristics of the participants (*N* = 1273)

Variable	Frequency	%
**Region**		
Shanghai	281	22.1
Zhejiang	505	39.7
Henan	487	38.2
**Age**^a^**(Years)**		
≤ 44	616	48.5
45–60	444	34.9
≥ 60	211	16.6
Missing	2	0.2
**Sex**^a^		
male	953	74.9
female	309	24.3
Missing	11	0.9
**Ethnicity**^a^		
Han	1218	95.7
Others	39	3.1
Missing	16	1.3
**Religious or not**^a^		
Yes	204	16.1
No	1063	83.9
Missing	6	0.5
**Employment status**^a^		
Employed or student	665	52.8
Unemployed or retired	595	47.2
Missing	13	1.0
**Marital status**		
Married	574	45.1
Single or divorced or widowed	699	54.9
**Time of treatment**^**a**^** (Years)**		
< 1	152	11.9
1–4	467	36.7
5–9	305	24.0
≥ 10	321	25.2
Missing	28	2.2

^a^Missing values are present

### Partial correlations among variables

The means, standard deviations, and partial correlations for all variables are presented in Table 2. Results showed that the independent variable HIV-related stigma was negatively correlated with the dependent variable MHRQoL (*r* = -0.207, *p* < 0.001). Depression (*r* = -0.351, *p* < 0.001) and anxiety (*r* = -0.495, *p* < 0.001) were negatively associated with MHRQoL. They were also negatively associated with social support (depression: *r* = -0.130, *p* < 0.001; anxiety: *r* = -0.130, *p* < 0.001). HIV-related stigma was significantly associated with depression (*r* = 0.088, *p* < 0.01), anxiety (*r* = 0.209, *p* < 0.001), and social support (*r* = -0.227, *p* < 0.001).

**Table 2:** Means, standard deviations and partial correlation coefficient between variables

Variables	Mean ± SD	HIV-related stigma	Depression	Anxiety	Social support	MHRQoL
HIV-related stigma	111.46 ± 15.21	1				
Depression	7.15 ± 2.43	0.088*	1			
Anxiety	5.81 ± 2.71	0.209**	0.495**	1		
Social support	59.52 ± 11.46	-0.227**	-0.130**	-0.130**	1	
MHRQoL	46.53 ± 10.20	-0.207**	-0.351**	-0.502**	0.100*	1

^*^Controlling for age, gender, religion, employment status *p < 0.01**p < 0.001

### Testing for mediation (anxiety as a mediator)

As shown in Table 3, the direct effect in the relationship between HIV-related stigma and MHRQoL was significant (B = -0.069, *p* < 0.001) as well as positively associated with anxiety (B = 0.037, *p* < 0.001). The indirect effect shown in the relationship between HIV-related stigma and MHRQoL via anxiety was also significant (B = -0.065, *p* < 0.001), indicating that anxiety also partially mediated the relationship between HIV-related stigma and MHRQoL.

**Table 3:** Regression results for simple mediation

Variable^a^	Coeff	SE	P	LLCI	ULCI
Depression regressed on HIV-related stigma	0.014	0.005	0.002	0.005	0.023
MHRQoL regressed on depression	-1.351	0.108	< 0.001	-1.562	-1.139
Effect of HIV-related stigma mediated by depression on MHRQoL					
Direct effect	-0.115	0.017	< 0.001	-0.148	-0.081
Indirect effect	-0.019	0.007	< 0.001	-0.033	-0.006
Total effect	-0.134	0.018	< 0.001	-0.169	-0.098
Anxiety regressed on HIV-related stigma	0.037	0.005	< 0.001	0.028	0.047
MHRQoL regressed on anxiety	-1.730	0.092	< 0.001	-1.910	-1.550
Effect of HIV-related stigma mediated by anxiety on MHRQoL					
Direct effect	-0.069	0.016	< 0.001	-0.101	-0.036
Indirect effect	-0.065	0.010	< 0.001	-0.085	-0.046
Total effect	-0.134	0.018	< 0.001	-0.169	-0.098

^a^Controlling for age, gender, religion, employment status

### Testing for mediation (depression as a mediator)

Table 3 shows the mediating role of depression in the relationship between HIV-related stigma and MHRQoL. HIV-related stigma was positively associated with depression (B = 0.014, *p* = 0.002) and negatively associated with MHRQoL (B = -1.351, *p* < 0.001). Depression partially mediated the relationship between HIV-related stigma on MHRQoL (B = -0.019, 95% CI: -0.033 ~ -0.006, *p* < 0.001); the total effect of HIV-related stigma on MHRQoL was -0.134 (95% CI: -0.169 ~ -0.098, *p *< 0.001).

### Testing for moderated mediation (anxiety as a mediator; social support as a moderator)

**Fig. 1:**
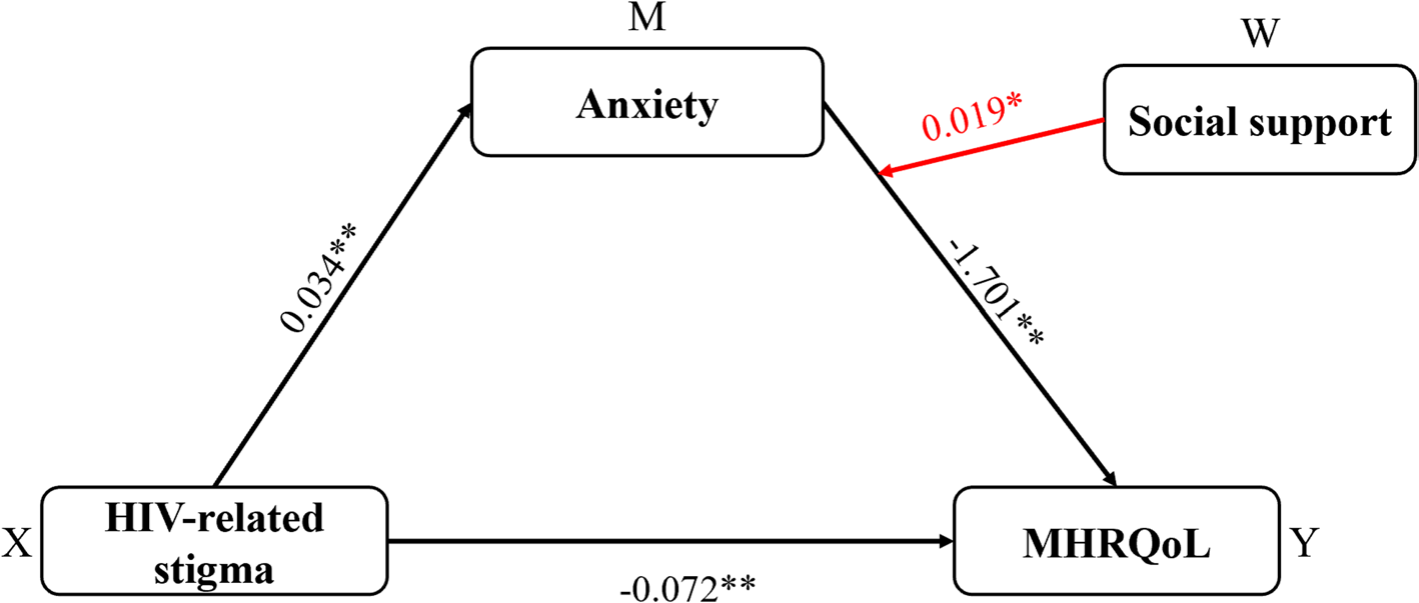
Statistical diagram of moderated mediation model 2 in the study. Note: Controlling for age, gender, religion, employment status *p < 0.01, ** *p* < 0.001

**Fig. 2:**
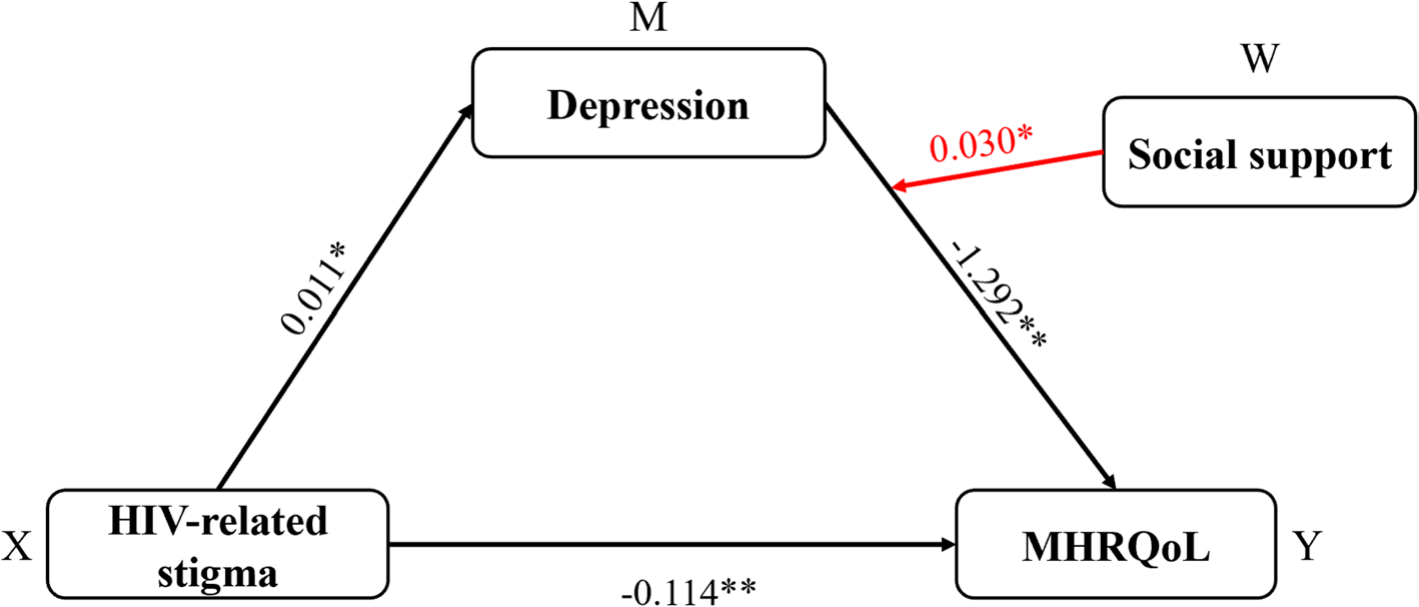
Statistical diagram of moderated mediation model 1 in the study. Note: Controlling for age, gender, religion, employment status *p < 0.05, ** *p* < 0.001

As indicated in Table 4 and Fig. 1, social support moderated the relationship between anxiety-mediated HIV-related stigma and MHRQoL (R^2^ = 0.345, *F* = 69.342, *p* < 0.001). Furthermore, the interaction between social support and anxiety was statistically significant for MHRQoL (B = 0.019, *p* = 0.007). The simple slope in Fig. 2 shows the moderating effect of social support on the relationship between anxiety and MHRQoL. Finally, as seen in Table 6, the indirect effect of anxiety in mediating HIV-related stigma on MHRQoL increased from -0.073 to -0.046 when social support increased from -11.412 (mean-SD) to 11.412 (mean + SD).

**Table 4:** Regression results for the conditional indirect effect (anxiety as a mediator)

Explanatory variables^a^	Anxiety				MHRQoL			
	B	SE	t	p	B	SE	t	p
Constant	-2.062	0.411	-5.019	< 0.001	53.859	1.323	25.248	< 0.001
HIV-related stigma	0.034	0.005	6.584	< 0.001	-0.072	0.017	-4.240	< 0.001
Anxiety					-1.701	0.092	-18.459	< 0.001
Social support	-0.020	0.007	-3.011	0.003	0.014	0.022	0.660	0.509
HIV-related stigma × social support	0.001	0.001	-0.905	0.365	0.001	0.001	1.311	0.190
Anxiety × social support					0.019	0.007	2.689	0.007
Model summary	R^2^ = 0.084 F = 15.627 *p* = < 0.001				R^2^ = 0.345 F = 69.342 *p* < 0.001			

^a^Controlling for age, gender, religion, employment status

### Testing for moderated mediation (depression as a mediator; social support as a moderator)

**Fig. 3:**
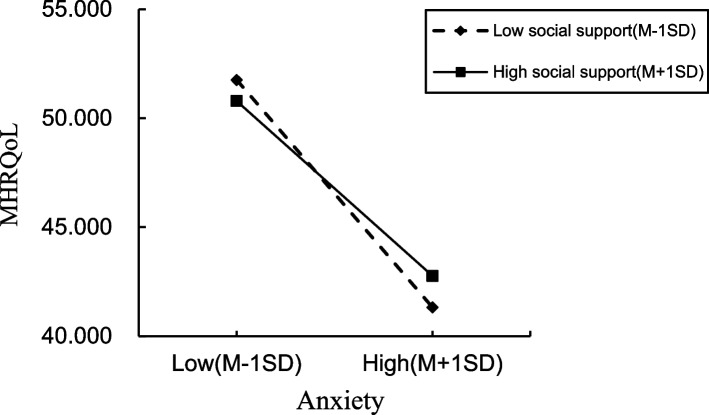
Association between anxiety and MHRQoL at higher and lower levels of social support

**Fig. 4:**
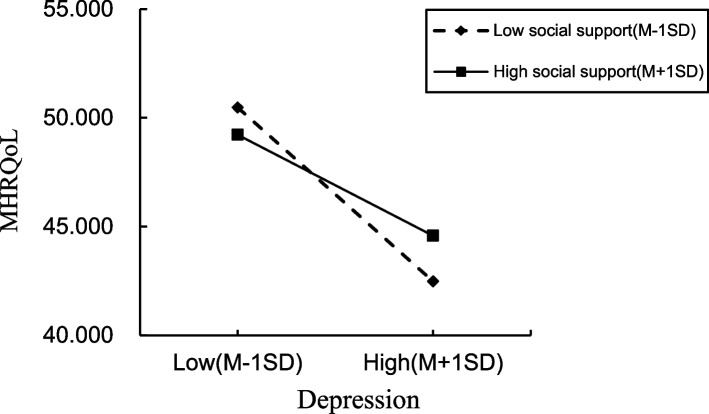
Association between depression and MHRQoL at higher and lower levels of social support

The results presented in Table 5 and Fig. 3 show that social support moderated the relationship between depression-mediated HIV-related stigma and MHRQoL (R^2^ = 0.250, *F* = 43.999, *p* < 0.001). Also as shown, the interaction with depression was statistically significant for MHRQoL (B = 0.030, *P* = 0.001). Figure 4 shows the moderating effect of social support on the relationship between depression and MHRQoL. Finally, the conditional indirect effect of HIV-related stigma on MHRQoL (via depression) was examined under three social support values (see Table 6), with effect values rising significantly from -0.025 to -0.006, but no significance demonstrated when the value was 11.412.

**Table 5:** Regression results for the conditional indirect effect (depression as a mediator)

Explanatory variables*	Depression				MHRQoL			
	B	SE	t	p	B	SE	t	p
Constant	-1.878	0.373	-5.034	< 0.001	54.739	1.408	38.885	< 0.001
HIV-related stigma	0.011	0.005	2.220	0.027	-0.114	0.018	-6.420	< 0.001
Depression					-1.292	0.109	-11.863	< 0.001
Social support	-0.024	0.006	-3.963	< 0.001	0.018	0.023	0.787	0.431
HIV-related stigma × social support	0.001	0.001	-1.406	0.160	0.001	0.001	1.368	0.172
Depression × social support					0.030	0.009	3.351	0.001
Model summary	R^2^ = 0.084 *F* = 15.627 *p* = < 0.001				R^2^ = 0.250 *F* = 43.999 *p* < 0.001			

^*^Controlling for age, gender, religion, employment status

**Table 6:** Conditional indirect effect of HIV-related stigma on MHRQoL at values of the moderator

Mediator	Moderator	Effect	Boot SE	Boot LLCL	Boot ULCL
Anxiety	-11.412	-0.073	0.016	-0.108	-0.045
	0.000	-0.059	0.010	-0.080	-0.040
	11.412	-0.046	0.010	-0.068	-0.026
Depression	-11.412	-0.025	0.011	-0.048	-0.006
	0.000	-0.014	0.007	-0.027	-0.001
	11.412	-0.006	0.006	-0.018	0.005

## Discussion

The negative impact of HIV-related stigma is pronounced in the Chinese cultural environment [36], but few previous studies have focused on the relationship between HIV-related stigma and MHRQoL. To delve deeper into these dynamics, this study developed a mediating model with moderating properties to examine the mediating role of depression/anxiety and the moderating role of social support in the relationship between HIV-related stigma and MHRQoL. The findings highlight that HIV-related stigma directly and adversely impacts MHRQoL, with greater severity of stigma leading to more pronounced harm to MHRQoL among PLWH. Additionally, anxiety was identified as a partial mediator in the association between HIV-related stigma and MHRQoL. Likewise, depression partially mediated this relationship. In exploring whether social support has an indirect effect on the relationship between HIV-related stigma and MHRQoL, the results support the hypothesized mediating model: the magnitude of the indirect effect depends on the degree of social support.

The present study demonstrated that anxiety was positively associated with HIV-related stigma and negatively associated with MHRQoL, mediating between the two variables. This finding is consistent with previous findings, for example, found that anxiety has a strong psychological impact on patients [37] and that anxiety and depression mediate the relationship between alexithymia and mental health [38]. The study not only reveals the negative impact of anxiety on the MHRQoL of the participants but also demonstrates that HIV-related stigma can further affect MHRQoL through its influence on anxiety. Thus, it is crucial to address the detrimental effects of HIV-related stigma and consider the MHRQoL of PLWH with anxiety, providing appropriate support to mitigate negative consequences.

As hypothesized, depression was shown to mediate the relationship between HIV-related stigma and MHRQoL, with a direct positive association with HIV-related stigma but a direct negative relationship with MHRQoL. In a study of incarcerated Malaysian men with AIDS, depression mediated the relationship between AIDS-related stigma and health-related quality of life [4]. These potential mechanisms of low self-esteem and negative self-image in PLWH may lead to worsening depressive symptoms and subsequent negative effects on mental health. This finding was confirmed in a previous study [39]. The preliminary exploration conducted in this study revealed that stigma significantly predicts MHRQoL [40]. It further emphasized the importance of personal engagement in negative emotion management, including anxiety and depression, as well as the reduction of public HIV-related stigma, as key factors for improving MHRQoL in people living with HIV (PLWH).

Extending the findings of previous studies, the study demonstrated a moderating effect of social support. Specifically, the negative impact of HIV-related stigma mediated through depression on MHRQoL was stronger in PLWH with less social support. This result is consistent with the finding that social support helps buffer the negative impact of depression on health-related quality of life [41]. However, in the present study this association was not significant among those with high social support.

Similarly, the presence of anxiety reinforced the negative impact of HIV-related stigma on MHRQoL but was mitigated in PLWH with high social support. Social support buffered the impact of anxiety-mediated HIV-related stigma on MHRQoL. This study also found that social support can directly affect anxiety and depression. This result is consistent with previous studies that found that social support may be important in improving depressive symptoms [42]. However, the direct effect on MHRQoL was not significant in this study, a finding that may be explained by the many factors that affect MHRQoL. Study findings indicated that social support plays a crucial role in improving MHRQoL in PLWH.Not only does it directly mitigate the negative impact of anxiety and depression associated with stigma, but it also moderates the tendency of negative emotions to further affect MHRQoL.

Thus, study findings highlighted the importance of considering the effects of stigma, anxiety, depression, and social support in efforts to improve MHRQoL in PLWH. Related psychotherapy research has found that stigma has a relatively powerful and long-lasting effect on well-being [43]; Reducing stigma can improve people’s mood, cognition, and behavior [44]. This study highlights that social support can mitigate the impact of HIV-related stigma on the MHRQoL of PLWH. It is crucial to provide social support from friends, family, and individuals to PLWH who face HIV-related stigma. Social support serves as a solid resource base, enabling PLWH to effectively navigate challenges and respond positively to stigma and discrimination in their daily lives [45]. Also, for PLWH who are already experiencing symptoms of anxiety/depression, the possibility of stigma or lack of social support is considered. In conclusion, addressing the adverse effects of HIV-related stigma and recognizing the moderating role of social support are essential for enhancing the MHRQoL of PLWH, especially for individuals with existing depression or anxiety. A multilevel systematic social support program should be developed based on the actual situation and needs of PLWH to effectively address these challenges.

### Strengths and limitations

Although this study advances the understanding of how psychosocial factors (HIV-related stigma, depression, anxiety, and social support) interact and influence MHRQoL, some research limitations must be considered. First, the study was a cross-sectional study with several drawbacks, including the inability to measure incidence, difficulty in making causal inferences [46], and the directionality of relationships. Second, some characteristics of the sample limited the generalizability of the findings. For example, the survey was conducted in three regions, Shanghai, Zhejiang, and Henan, and was not representative of the national situation; differences in age, economy, and culture may limit the extension of the findings. Third, some of the items on the SF-12 scale (e.g., do you feel overwhelmed at work or in daily activities because of your mood?) may indicate symptoms of depression and anxiety as reported on the HADS scale. This overlap may have had implications for the overall mediating model of adjustment tested in the study.

Despite these limitations, the present study had some advantages. The study had a large sample size, which enhanced the statistical effects of the exploration. The study went beyond the exploration of the HIV-related stigma mechanisms affecting MHRQoL to consider the moderating role of social support in the model. Finally, the study provides a reference for follow-up studies and further investigation of MHRQoL of people living with HIV.

## Conclusion

The current study established that the partial mediating role of anxiety and depression in the relationship between stigma and MHRQoL among people living with HIV in China was moderated by social support. The results suggested that depression, anxiety, and social support play important roles in MHRQoL of PLWH. Future interventions that aim to improve MHRQoL of PLWH should have primary goals that include reducing stigma, anxiety, and depressive symptoms, as well as increasing social support.

## Data Availability

Due to privacy constraints, the datasets generated and analyzed in this study are not publicly available but are available from the corresponding author on reasonable request.
